# Vitamin E and the Healing of Bone Fracture: The Current State of Evidence

**DOI:** 10.1155/2012/684510

**Published:** 2012-12-09

**Authors:** Boekhtiar Borhanuddin, Nur Farhana Mohd Fozi, Isa Naina Mohamed

**Affiliations:** Pharmacoepidemiology and Drug Safety Unit, Department of Pharmacology, Faculty of Medicine, National University of Malaysia, Jalan Raja Muda Abd Aziz, 50300 Kuala Lumpur, Malaysia

## Abstract

*Background.* The effect of vitamin E on health-related conditions has been extensively researched, with varied results. However, to date, there was no published review of the effect of vitamin E on bone fracture healing. *Purpose.* This paper systematically audited past studies of the effect of vitamin E on bone fracture healing. *Methods.* Related articles were identified from Medline, CINAHL, and Scopus databases. Screenings were performed based on the criteria that the study must be an original study that investigated the independent effect of vitamin E on bone fracture healing. Data were extracted using standardised forms, followed by evaluation of quality of reporting using ARRIVE Guidelines, plus recalculation procedure for the effect size and statistical power of the results. *Results.* Six animal studies fulfilled the selection criteria. The study methods were heterogeneous with mediocre reporting quality and focused on the antioxidant-related mechanism of vitamin E. The metasynthesis showed **α**-tocopherol may have a significant effect on bone formation during the normal bone remodeling phase of secondary bone healing. *Conclusion.* In general, the effect of vitamin E on bone fracture healing remained inconclusive due to the small number of heterogeneous and mediocre studies included in this paper.

## 1. Introduction

According to Castellini et al., vitamin E has been regarded as “the most potent, lipid-soluble, chain-breaking antioxidant in nature” [1]. There are eight distinct, natural forms of vitamin E: four tocopherol isomers (*α*, *β*, *γ*, and *δ* isomers) and four tocotrienol isomers (*α*, *β*, *γ*, and *δ* isomers) [2]. Of all these isomers, *α*-tocopherol has been the most studied because it has the highest bioavailability in human tissues [3]. It is seen as an antioxidant that is required for the protection of cell membranes, in which it reacts with free radicals (i.e., peroxyl and alkoxyl radicals) to preserve polyunsaturated fatty acids in the membrane structure [4]. Besides that, *α*-tocopherol has also been investigated for its role in the activation of several genes [5]. 

Nevertheless, there is a dearth of investigation on the biological activity of other isomers of vitamin E, especially in humans. Moreover, the studies of the effect of vitamin E on health-related conditions have been vast and with varied results [2]. Recently, a new controversy has entered into the picture, whereby *α*-tocopherol was purported to decrease bone mass, through the simulation of osteoclast fusion [6]. This study has prompted us to reexamine the relationship between vitamin E and bone-related conditions. Our focus is on the effect of vitamin E on bone fracture healing. Based upon the initial background search on the issue at hand, the most popular proposed mechanism of action for the effect of vitamin E on bone fracture healing is based upon the cellular-protective property of antioxidant. In bone fracture, oxygen free radicals are produced by the activation of polymorphonuclear neutrophils in the inflammatory phase of bone fracture healing [7], as well as by the impairment of blood supply to the bone ends [8]. These free radicals have been shown to inhibit bone fracture healing [9, 10] by initiating a chain reaction that will cause lipid peroxidation that leads to cell membrane damage and eventually cell lysis [7]. The protective role of vitamin E in this situation may be seen in past studies, where *α*-tocopherol in the cell membranes was shown to act as an antioxidant that inhibits lipid peroxidation by scavenging the free radicals and thus breaks the chain reaction [11, 12]. However, there was at least one study that reported *α*-tocopherol did not have any effects on the lipid peroxidation and antioxidant enzyme activities [13].

Preliminary search in the major databases (MEDLINE, CINAHL, and Scopus) in early March 2012 yielded no result for any narrative reviews, systematic reviews, or meta-analyses of the effect of vitamin E on bone fracture healing. Hence, a proper systematic review will provide a critical evaluation of the current state of evidence for this topic. Additionally, this paper will also serve as a methodological guideline for other researchers who would like to pursue this line of investigation. Generally written in a methodological auditing manner, this systematic review has attempted to fulfil the following seven specific objectives:

- brief description of general study designs of past studies of the effect of vitamin E on bone fracture healing;
- brief description of the results from past studies of the effect of vitamin E on bone fracture healing;
- brief description of the suggested mechanism of action for the effect of vitamin E on bone fracture healing in past studies;
- evaluation of the quality of the reported methods and results from past studies of the effect of vitamin E on bone fracture healing;
- evaluation of the effect size and statistical power of the effect of vitamin E on bone fracture healing in past studies;
- calculation of optimum sample size for replication of past studies of the effect of vitamin E on bone fracture healing;
- metasynthesis of specific patterns about the effect of vitamin E on bone fracture healing.

## 2. Methods

In line with the current emphasis on the reduction of bias in concluding the evidence in studies [14], the recommended steps for a systematic review were followed, with a few minor adjustments. An extra step was added in this paper, whereby the data in the included studies were reanalysed, if necessary and possible. This is to provide a better picture of the conclusion derived from the studies.

### 2.1. Study Selection

**Figure 1:**
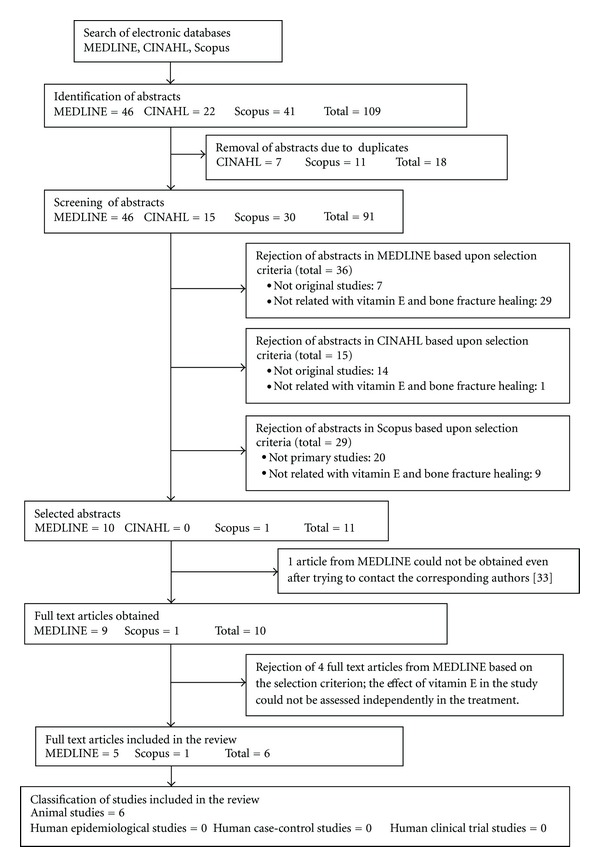
Flowchart to show the selection process of the articles in this review.

A systematic electronic search was conducted in three databases (MEDLINE, CINAHL, and Scopus), encompassing a publication timeline from 1946 to the end of March 2012. The search strategy included the following terms and Boolean operators: (1) vitamin E OR Vit* E OR tocotrienol OR tocopherol; (2) fracture* OR bone* healing; (3) 1 and 2 (all fields; limit to English language only). The search strategy was created by a panel of two researchers with mutual consensus. Its focus was more on sensitivity, rather than specificity, in order to identify as many articles as possible, regardless of the type of study. Due to the lack of resources, there was no effort to include non-English language studies, or to attempt hand-searching the related abstracts. Via this strategy, a total of 109 individual hits were identified (Figure 1). All abstracts from the hits were then downloaded, while 18 duplicates were removed in the process. The pooled 91 abstracts were then screened using a standardised electronic form by two researchers independently. At this stage, abstracts were included in the review based upon these following criteria: (a) the study reported the effect or association between vitamin E isomer and the healing of bone fracture-related conditions; (b) the fracture-related condition was related to lifestyle variables, aging or experimentally-induced conditions. At the same time, abstracts were excluded from the review, if (a) the study was not an original study (e.g., review, letter, or editorial), or (b) the study was related to bone fracture secondary to other pathologies (besides the ones stated in the inclusion criteria). Ambiguous abstracts were excluded only after their full papers were traced and screened in the next phase. Any discrepancies in the results of the screening were resolved through a consensus process. The abstract screening yielded 11 abstracts that fulfilled the selection criteria. This was followed by tracing the full articles for each abstract electronically and manually. These articles were then subjected to the final round of screening by two researchers using a standardised electronic form independently. In this phase, the study would be rejected from the review, if the effect of the vitamin E on bone fracture healing could not be assessed independently (i.e., the vitamin E was mixed with other active compounds). Any disagreements in the screening results were settled by mutual consensus. Via this process, only six full articles were finally included in the review, of which all were animal studies. Due to the small number of the pooled full articles, all studies identified in this phase were included in this review, regardless of their quality of evidence. Nevertheless, to reduce the chance of making a flawed conclusion, the quality of each article was evaluated as a part of the data extraction process.

### 2.2. Data Extraction

Data extraction procedure was performed by a single researcher, followed by foolproofing by another researcher. Data extraction for each included studies was performed using three different electronic forms—the study design form, the study results form, and the study report quality form. In the study design form, the following information was recorded: (a) sample characteristics; (b) affected bone(s); (c) fracture production procedure; (d) fracture type; (e) fracture fixation/distraction procedure; (f) dose of vitamin E isomer(s) used in the treatment group; (g) duration of treatment; (h) intervention in the control and other comparison group(s); (i) main outcome measure(s). On the other hand, the study results form required the researcher to record the following information: (a) reported descriptive values of main outcome measure(s); (b) reported inferential statistical analysis; (c) result summary; (d) model proposed for the mechanism of action of vitamin E in the study; (e) conclusion of the effect of vitamin E on the phase of fracture healing reported in the study. Finally, the quality of the study report was assessed using a checklist based upon the Animal in Research: Reporting In Vivo Experiments (ARRIVE) Guidelines. For this paper, only 22 individual items in Section 2 (item 5 through item 13c) and 6 items in Section 3 (item 14 through item 17b) were used. The ARRIVE Guidelines were specifically developed to provide an outline to optimise the information provided in publications of animal studies, through high-quality and comprehensive reporting [26].

### 2.3. Reanalysis of Study Results

To reduce the element of subjective judgement in drawing the conclusion, an extra phase was included in the review process. In this phase, the reported results focusing on the comparison of outcome parameters between the vitamin E treatment group and the control group would undergo statistical reevaluation by an assigned researcher and later would be confirmed by another researcher. Using an electronic form, specific commentaries were given on each relevant statistical analysis reported in the articles. This was followed by a reanalysis of the relevant reported data, as well as the calculation of effect size, statistical power, and sample size for replication studies. A reanalysis of the data was performed using the software Statistical Package for the Social Sciences (SPSS) Version 16.0. In this paper, this had been done by reanalysing the cross-tabulated count-based outcomes reported in the articles via Mann-Whitney *U* test, when it was necessary and possible. Meanwhile, the effect size, statistical power, and sample size calculation were performed using G* Power Version 3.1.2. These calculations were only performed if the mean and standard deviations of the outcome parameters were available, either in the report or through the reanalysis procedure. The standardised effect size (Cohen's *d*) and statistical power calculations were performed based on the alpha value set at 0.05 (two tailed) with minimum asymptomatic relative efficiency (ARE) distribution (i.e., a type of distribution for nonparametric test) [27]. The sample size calculation for replication study was also performed based on the previous alpha value and type of distribution, as well as the statistical power set at 0.80. 

## 3. Results

### 3.1. Brief Description of General Study Designs of Past Studies of the Effect of Vitamin E on Bone Fracture Healing

One of the most prominent findings in this systematic review was the absence of any published human studies, neither epidemiological observation, case-control study, nor clinical trial, on the association or effect of vitamin E on bone fracture healing. Besides that, another salient observation was the extreme heterogeneity of the study methods employed in the articles included in this paper. Of the six experimental-based animal studies published (Table 1), three studies were based upon the rat model [18, 21, 24], two were based upon the rabbit model [15, 23], and only one was based upon the dog model [8]. The bone model being investigated in the studies was primarily normal bone, except one study that focused on osteoporotic bone model [18]. One of three types of fracture production procedure was generally used in these studies: osteotomy [8, 15], manual fracture [21, 23, 24], or 3-point bending method using a guillotine device [18]. Nearly all of the studies were based upon a secondary bone healing model promoted by either internal fixation using Kirschner wires [18, 21], external fixation [15, 23], or left *in situ* [24]. Only one study was based upon primary bone healing model promoted by internal fixation using plates [8]. To date, there was no published study of the effect of tocotrienol analog on bone fracture healing. All these studies used *α*-tocopherol isomer for their treatment group, with the dose ranging from 20 to 100 mg/kg/day for the duration between 5 to 60 days. Only one study administered the *α*-tocopherol treatment for a short duration, before stopping it while the experiment was still going on [24]. Other studies administered the *α*-tocopherol treatment continuously until the end of their experiments. The *α*-tocopherol treatment group would generally be compared to a control group that received no treatment at all or only received normal saline/vehicle. Only two studies deviated from this general design. One study observed the effect of the treatment of *α*-tocopherol on osteoporotic bone fracture compared to normal bone and osteoporotic bone control groups [18]. The other study compared the *α*-tocopherol treatment group against a control group, a vitamin C treatment group and a combination treatment (*α*-tocopherol and vitamin C) group [24]. Besides that, there was also heterogeneity in the main outcome measures of each study. These outcomes may be clustered into five major groupings: (a) radiological-based assessment of bone formation [8, 15, 18, 21]; (b) histological-based assessment of bone formation [15, 21, 23, 24]; (c) radiological-based assessment of callous volume/index [18, 24]; (d) radiological-based assessment of callous formation [8, 18]; (e) osteoblastic activity assessment [15].

### 3.2. Brief Description of the Results from Past Studies of the Effect of Vitamin E on Bone Fracture Healing

Based on Tables 2 and 4, nearly all the studies reported their inferential results based on nonparametric statistical analyses, using either Mann-Whitney *U* test, Kruskal-Wallis test, or chi-square test. There was only one study that did not report the results of the statistical analysis, although the overall conclusion of the *α*-tocopherol treatment effect was reported [8]. Disregarding the heterogeneity of the studies, a significant superior effect of the *α*-tocopherol treatment group compared to the control was seen in the radiological-based bone formation parameter [8, 15, 18, 21] and in the histological-based bone formation parameter [15, 21, 23]. In terms of the assessment of callous-related parameters, most of the related studies showed no significant difference between the *α*-tocopherol treatment group compared to the control group [18, 24], except in one study [8]. A significant superior effect of the *α*-tocopherol treatment group compared to the control group was also seen in the osteoblastic activity parameter in one study [15]. However, one study [24] demonstrated that there was a significant inferior effect of the *α*-tocopherol-only treatment group compared to the vitamin C-only treatment group, in terms of the bone formation parameters and callous-related parameters. In the same study, the *α*-tocopherol-only treatment group was also significantly inferior to the vitamin C and *α*-tocopherol combination group, which in turn was significantly inferior to the vitamin C-only group, in terms of the aforementioned parameters.

At face value, it is tempting to generalise that most of the evidence showed that *α*-tocopherol is beneficial for bone fracture healing. However, this would not take into account the quality and the weight of evidence in each study. A better look at these issues would be done later on in this section. Additionally, the significant inferior results of *α*-tocopherol when compared to vitamin C, as well as the combination of vitamin C and *α*-tocopherol, will only be touched in Section 4.

### 3.3. Brief Description of the Suggested Mechanism of Action for the Effect of Vitamin E on Bone Fracture Healing in Past Studies

**Figure 2:**
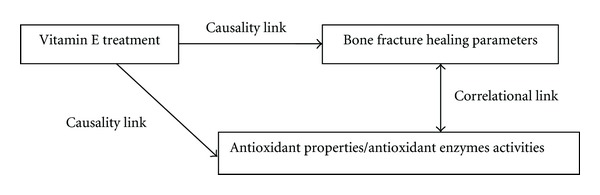
The framework of the general study design of the proposed mechanism of action of vitamin E on bone fracture healing in the included studies.

Out of the six studies included in this paper, only four studies reported the testing of their proposed mechanism of action for vitamin E effect on bone fracture healing [8, 15, 18, 21] (Table 2). In general, two suggested mechanisms were tested in these studies: (a) the positive effect of antioxidant property of vitamin E on bone fracture healing [15, 21] and (b) the positive effect of vitamin E on antioxidant enzymes activities, which in turn will have a positive effect on bone fracture healing [8, 18]. The parameter of the antioxidant property of vitamin E was measured as Total Antioxidant Capacity level [15] or Erythrocyte Malondialdehyde level [21]. On the other hand, the parameter of the antioxidant enzymes activities was measured via Bone Superoxide Dismutase level [18], Bone Glutathione Peroxidase level [18], or Serum Catalase level [8]. All four studies reported that their proposed mechanism was supported by their findings, through the concurrent increase of the previously mentioned parameters during the improvement of bone fracture healing. However, this conclusion is debatable as the experimental design in these studies did not support a causality link between the increase of the antioxidant-related parameters with the improvement of bone fracture healing. This is clearly illustrated in Figure 2, whereby the proposed mechanism was tested through a quasi-experiment design that would only support a correlational link between the mechanism and the improvement.

### 3.4. Evaluation of the Quality of the Reported Methods and Results from Past Studies of the Effect of Vitamin E on Bone Fracture Healing

Based upon the ARRIVE Guidelines, the total items that were described satisfactorily in the reviewed articles ranged between 8 to 13 per 22 items in Section 2 and between 0 to 3 per 6 items in Section 3 (Table 3). All included articles fulfilled less than half of the recommendations in Sections 2 and 3. Of particular interest, only three studies mentioned the use of randomisation during the allocation of experimental groups [8, 15, 18] and only two studies reported the use of blinding during the assessment of results [15, 18]. The absence of these components may reflect the probability of subjective bias in the study design itself [28]. Besides that, unsatisfactory description of the characteristics of the animal subjects as well as their housing was present in nearly all the reviewed articles. For example, some studies failed to report the baseline data, sex, age, and source of the animal subjects. Additionally, none of the reviewed articles reported any sample size calculations for their experiments or any attempts of independent replications of the results. As can be seen in Table 4, two studies did not use the appropriate inferential analysis that may yield a more conclusive result [18, 21]. In these studies, the chi-square test was used instead of the Kruskal-Wallis test or Mann-Whitney *U* test. Besides that, one study did not even bother to report the quantitative results (both descriptive and inferential), aside from a highly questionable statement that the results were significant [8]. 

### 3.5. Evaluation of Effect Size and Statistical Power of the Effect of Vitamin E on Bone Fracture Healing in Past Studies

Referring to Table 4, reanalysis was performed (where necessary and possible) primarily to provide the needed descriptive data for the effect size and statistical power calculation using G* Power software. Among the significant results, only one radiological-based assessment of bone formation from one study [18] had a statistical power value of 0.49. This value was far below the accepted conventional value of 0.80 [29]. On the other hand, the effect size of *α*-tocopherol treatment for (a) radiological-based assessment of bone formation ranged from 0.56 to 2.50 [15, 18, 21]; (b) histological-based assessment of bone formation ranged from 1.49 to 2.46 [15, 21, 23]; (c) radiological-based assessment of callous formation ranged around 0.15 [18]; (d) osteoblastic activity assessment ranged around 0.47 to 2.35 [15]. Conventionally, a Cohen's *d* value of 0.20 denotes a small effect size; a value of 0.50 denotes a medium effect size, whereas a value of 0.80 denotes a large effect size [30]. Therefore, the wide range of effect sizes of *α*-tocopherol implied that it is too simplistic (and misleading) to summarise the vitamin E effect from a single angle. It would be more prudent to examine the effects separately in different situations, procedures, or even models. The identification of the common core elements and patterns was attempted later, through metasynthesis [31]. 

### 3.6. Calculation of Optimum Sample Size for Replication of Past Studies of the Effect of Vitamin E on Bone Fracture Healing

The calculated optimum sample size for each significant result ranged between 5 to 16 subjects for each experimental group (Table 4). Generally, most of the studies used enough subjects for the testing of each main outcome measure [15, 21, 23]. However, one study did not have enough sample size for a good statistical analysis with proper statistical power [18]. In this study, the sample size for each group was five subjects, rather than 16 subjects as calculated. This indicates a higher chance for a nonsignificant result, if this study is replicated in the future.

### 3.7. Metasynthesis of Specific Patterns about the Effect of Vitamin E on Bone Fracture Healing

The conclusion about the effect of vitamin E would be incomplete, if there was no dissection on the results based upon the various study methods, models, or phase of bone healing. After several exploratory cross-tabulations of the results according to different sets of categories, several significant patterns can be seen, as shown in Table 5. Only the results of the comparison between the vitamin E treatment group and the control group were included in this table. One study [8] was omitted because of the lack of any quantitative results. Based on the table, the significant and positive effect of *α*-tocopherol was consistently observed after day 30 post-fracture, for all types of assessment of bone formation. These results had a large effect size and acceptable statistical power. However, there were mixed results between day 20 to day 29 postfracture for all types of assessment of bone formation. During this phase, the significant and positive effect of *α*-tocopherol was only observed in the histological-based assessment of fractures stabilized by cast immobilization (with a large effect size and acceptable statistical power) [23]. There were also mixed results for all types of assessment of bone formation between day 10 to day 19 post-fracture. During this phase, the significant and positive effect of *α*-tocopherol was only observed in the radiological-based assessment of fractures stabilized by internal fixation using the Kirschner wire [18]. Although this result showed a large effect size, the statistical power was unacceptable. It is also of interest to note that this study used a much higher dose of *α*-tocopherol (i.e., 60 mg/kg/day per oral), if compared to other studies that typically used a dose of 20 mg/kg/day intraperitoneally or intramuscularly. A rather startling pattern was seen, in terms of the effect of *α*-tocopherol on the callous-related assessments; virtually none of the results was significant, regardless of the day of observation [18, 24]. Likewise, none of the results related to the study where the fracture was left *in situ* was significant, either via bone formation assessments or callous-related assessments [24]. However, it must be remembered that this particular study administered the *α*-tocopherol treatment only in the early phase of the experiment and discontinued it for the rest of the study period. Besides that, the significant and positive effect of *α*-tocopherol on osteoblastic activity was seen between day 20 to day 29 post-fracture, but not earlier on [15].

In this metasynthesis, the noncommittal terms of “early phase” or “late phase” of bone healing reported in most of the reviewed articles will be ignored. The absence of a definite cut-off point for the bone healing timeline will hamper the understanding of the underlying mechanism of action. Besides that, the timeline of the phase of bone healing in rabbits was assumed to be nearly similar to rats, for the sake of simplicity. For secondary bone healing, callous formation is at the maximum around day 14 post-fracture, whereas the peak of bone remodeling phase occurs around day 28 to day 35 post-fracture in rats [32]. As the callous lasted from day 3 to day 30 post-fracture, there is an overlap with the bone remodeling phase that started as early as day 14 post-fracture. Once the recategorisation of the bone healing phase was done based on these cut-off points, the whole picture becomes clearer. Four specific patterns may be derived from the evidence in Table 5: (a) there was a significant and positive effect of *α*-tocopherol on bone formation during the peak of bone remodeling phase of secondary bone healing in animal models; (b) there were mixed results on the effect of *α*-tocopherol on bone formation during the callous formation phase of secondary bone healing in animal models; (c) there was no significant effect of *α*-tocopherol on callous formation itself during secondary bone healing in animal models; (d) there was a significant effect of *α*-tocopherol on the osteoblastic activity starting from the later stage of callous formation (which overlaps with the beginning of bone remodeling phase) of secondary bone healing, but not in the earlier stage of callous formation in animal models. 

## 4. Discussion

### 4.1. The Issue of the Lack of Human Studies in Investigating the Effect of Vitamin E on Bone Fracture Healing

To date, there was no published human study of the effect of vitamin E on bone fracture healing. This is perplexing, considering that the first related animal study was published back in 1996 by Durak et al., who reported a favourable *α*-tocopherol effect on fracture haematoma [33]. This lack of interest might be partially fuelled by the damaging evidence shown by one of the early animal study papers on this topic. The study by Saris$\ddot{\text{o}}$zen et al. gave a rather convincing evidence that *α*-tocopherol has a significantly inferior effect on bone fracture healing, if compared to vitamin C and the combination of vitamin C and *α*-tocopherol [24]. As the effect of vitamin E compared to control was also not significant in this study, these results might cause a dilemma in potential researchers. It seemed to be a safer bet to pursue vitamin C studies for the promotion of bone fracture healing. Furthermore, the fact that the combination group had significantly inferior effect to vitamin C, suggested a form of negative interaction between the two vitamins. Apparently, the dominant effect of vitamin C was reduced by the addition of *α*-tocopherol. However, we have to bear in mind that this was the result of only a single study, without any following attempts for independent replications. The total reliance on a popularised single study to reflect “the truth” may lead to “herding” behaviour among researchers [34]. They might decide to follow the path of investigations of popularised publications, while neglecting novel and independent research that may bear better fruits.

The importance of human studies in determining the effect of vitamin E on bone fracture healing cannot be denied. According to the 2009 classification by the Oxford Centre for Evidence-based Medicine, the highest level in the hierarchy of evidence is reserved for systematic reviews of randomised controlled clinical trial in humans [35]. According to one current view, animal studies lie at the lowest rung in the hierarchy of evidence for therapeutic intervention [36], at the same level with unsystematic anecdotal information or expert opinion. Therefore, if there was a lack of studies in the lowest level in the hierarchy, the decision to proceed to the next level might be affected. For example, researchers in the United Kingdom who are planning clinical trials are required to show that there are systematic reviews of previous related studies [37]. Besides that, scepticism has risen about the contribution of animal studies towards beneficial findings in humans. According to Pound et al., methodological flaws that were rampant in many animal studies would render the findings inconclusive [37]. They called for an urgent formal evaluation to address the actual contribution of animal studies to clinical medicine. According to them, it is still uncertain whether even valid results from animal experiments may be generalised to human beings.

### 4.2. The Issue of Reporting Quality of Reviewed Articles

As reported in Section 3, the quality of reporting of all the reviewed papers was mediocre, if the ARRIVE Guidelines were meticulously followed. Of specific interest is the lack of blinding and randomisation procedures reported in the majority of these articles. It should be noted that animal studies that did not report randomisation and blinding were more prone to report significant difference of their study groups, if compared to studies that used these bias-reducing methods [38]. The main purposes of the guidelines were to guarantee that the article included all relevant information for in-depth critique via the peer-review process [26], as well as to allow the animal experiment to be replicated [39]. Nevertheless, the quality assessment performed on the report of a study might not actually reflect the quality of the study* per se*. There was a possibility that the authors neglect to report the important information, due to ignorance or carelessness. Notwithstanding of this apologetic stand on behalf of the affected authors, it is predicted that there might be some problems in independently replicating the studies in this current paper. Difficulties might arise, if independent researchers totally rely on the reported version without knowing the exact methods through contact with the authors of the studies. In a recent scathing commentary on the state of preclinical cancer research, Begley and Ellis reported that only 11 percent of “landmark” studies (6 out 53 studies) could be reproduced, in terms of their findings [40]. These failures of study replication were partially blamed on technical differences and difficulties, as well as the pressure to publish a “perfect story” for the studies. It was stated that some of these nonreproducible papers had “spawned entire fields” of study. The materials of these papers were expanded by hundreds of subsequent secondary publications, without any attempts to confirm the original observations. Another prominent issue in the current reviewed articles was the absence of any attempts to describe how the sample size was actually derived. The rational selection of group size via pilot study or power analysis has been suggested by the Institutional Animal Care and Use Committees in the United States as a way to ensure researchers have enough subjects to reach their study objectives without wasting animals [41]. This is in conjunction with the use of appropriate statistical tests to generate the maximum information from the minimum number of animal subjects. This optimal use is performed in the spirit of the Reduction principle of “3 Rs” tenets of humane treatment of experimental animals, as originally proposed by Russell and Burch in 1959 [42]. The other 2 Rs are Replacement and Refinement. For the reviewed studies, it was unknown whether the sample size determination was performed using *a priori* power analysis, or based upon some pilot test results. There is also a possibility that they just depended on some fixed, arbitrary rule of accepted sample size number in their respective institutions. It should be stressed here that a reduction of animal sample size might not be justified, if the experiment does not have enough statistical power to detect any meaningful effect.

### 4.3. The Issue of Explaining the Actual Effect of Vitamin E on Bone Fracture Healing

There are several problems of drawing a valid conclusion on the effect of vitamin E on bone fracture healing based upon the heterogeneous findings of the reviewed articles. These difficulties could be summarised by the list of methodological problems of animal experiments as highlighted by Pound et al. [37]. Among their exhaustive list, four problems essentially present in this current systematic review: (a) dissimilar animal species with different metabolic pathways, which may lead to variations in efficacy; (b) dissimilar models for inducing the injury or illness, which may vary in human condition; (c) variability in the selection process, randomisation procedure, and choice of comparison treatment(s); (d) obscurity of the laboratory techniques that may affect results, such as blinding procedures. In spite of this, the four patterns of results showed in the meta-synthesis could be further simplified into a single statement: *α*-tocopherol may promote bone formation during the peak of the bone remodeling phase of secondary bone healing in animal models, but not callous formation. The *α* -tocopherol's effect on bone formation during the late callous formation phase remained inconclusive, due to the mixed results that were observed during this phase. These inconsistencies might be related to its overlap with the start of the bone remodeling phase. Besides that, the single result that showed a significant effect for bone formation during the early callous formation phase [18] might be disregarded as an outlier. This is due to its low statistical power, which reflects lower reproducibility likelihood. 

**Figure 3:**
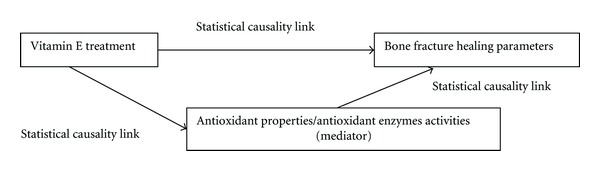
The framework of meditation analysis for the proposed mechanism of action of vitamin E on bone fracture healing in the included studies.

Does this generalisation on the *α*-tocopherol's effect give any credibility to the postulated antioxidant-based mechanism of action described earlier on? None of the reviewed studies was properly designed and analysed to test the causality link of the antioxidant-based mechanism of action of vitamin E on bone fracture healing (Figure 2). This weakness may be solved, if the researchers actually used a more sophisticated statistical analysis called mediation analysis with the proper use of dummy variables [43–45]. Through this analysis, it is possible to test the statistical causality link between the vitamin E treatment and bone fracture healing parameters, with the postulated mechanism parameter as a mediator (Figure 3). If the mediation is significant, the researchers may confidently suggest that mechanism of action is likely to be the cause of the change in the bone fracture healing parameter. However, the failure of the reviewed studies to employ this test has jeopardised the chance of testing the mechanism theory directly in the studies. This failure is eerily reminiscent of the situation warned by Begley and Ellis, whereby many secondary investigations were performed based upon expansion of the materials of unsubstantiated original observation [40]. 

### 4.4. Implications and Suggestions for Future Studies

Based upon the fact that there were only six related published animal studies in major databases on the topic of bone fracture healing and vitamin E, it is much too early to endorse a more general conclusion. Nevertheless, this limited evidence looked favourable for vitamin E, at least for the promotion bone formation. There are still plenty of rooms for new studies to fill in the various gaps in the evidence as seen in the meta-synthesis shown in Table 5. These new studies may be designed according to other experimental conditions that have not yet been studied, such as the testing of the tocotrienols. There is also merit in designing proper studies to describe conclusively the biochemical mechanism behind the action of vitamin E in bone fracture healing. Some efforts should also be put to replicate the original studies and the subsequent secondary studies to reduce the chance of making a premature false conclusion based on irreproducible studies. In view of the state of evidence in this paper, it is cautiously recommended that investigators begin venturing into observational studies in humans. It is important to see whether the positive findings seen in animal studies might be also true for human beings. For example, cross-sectional studies may be performed on the correlation between overall vitamin E intake in the daily diet and the speed of bone fracture healing among orthopaedic patients. Any positive findings in observational studies might then hasten the progress for proper clinical trials. This methodological upgrade will determine the actual causal effect of vitamin E on human bone fracture healing. Besides that, future studies should take note on the current guidelines of quality reporting to ensure that their results would be reproducible by other independent researchers. On top of that, the issue of sample size justification must not be taken for granted, as there are now established methods or how to handle this optimally. These methods may also be applied to human studies. On the other hand, any attempts to confirm the mechanism of action of vitamin E effect should either use the proper experimental designs or at least employ the proper mediation analysis method.

### 4.5. Limitation of This Systematic Review

As in many other systematic reviews, this current paper was not immune to the effect of publication bias [28]. This bias happened because there is a systematic exclusion of many studies with nonsignificant results that were not published in peer-reviewed journals. Besides that, the absence of hand-searching for original studies not included in the searched databases, as well as the exclusion of non-English language articles, might have caused some studies to be systematically left out [14, 46]. 

## 5. Conclusion

Based on the small number of heterogeneous and mediocre animal studies included in this systematic review, the effect of vitamin E on bone fracture healing remained inconclusive. Despite this, the effect of *α*-tocopherol on bone formation during the normal bone remodeling phase of secondary bone healing in the reviewed studies was promising; although it has not been shown be effective for callous formation It is recommended that more original studies (experimental animal studies, especially basic mechanism research, and human observational studies) to be properly performed on this topic. These efforts will help in the formulation of a more comprehensive generalisation on the status of vitamin E in bone fracture healing, particularly for humans.

## Figures and Tables

**Table 1:** Evidence table for past animal studies of the effect of vitamin E on bone fracture healing.

Study	Sample (size of study groups)	Study design							
		Affected bone(s)	Fracture production	Fracture type	Fracture fixation/distraction	Dose of vitamin E analog used in treatment group	Duration of treatment	Intervention in control and other comparison group(s)	Main outcome measure(s)
Kurklu et al. [15]	Adult New Zealand white rabbits (control = 15; treatment = 15)	Middle third of right tibia (normal bone model)	Osteotomy	Open fracture	Distraction using circular external fixator	20 mg/kg/day *α*-tocopherol (intramuscular)	30 days	Control group did not receive any treatment.	Radiological evaluation of bone formation (based upon the grading system by Lane and Sandhu [16]) on day 20, day 30, and day 40 of study Scintigraphical assessments to calculate the osteoblastic activity ratio (distracted tibia/contralateral normal tibia) on day 5 and day 20 of study Histopathological evaluation of bone formation of the distracted tibial segments (based upon the grading system by Huddleston et al. [17]) on day 40 of study

Shuid et al. [18]	Female Sprague-Dawley rats (sham = 8; control = 8; treatment = 8)	Mid-diaphysis of right femur (osteoporotic bone model)	Three-point bending method using a guillotine device	Closed fracture	Internal fixation using Kirschner wires	60 mg/kg/day of *α*-tocopherol (per oral gavage) (ovariectomized treatment group)	14 days	Sham-operated and ovariectomized control groups received vehicle only (oral gavage)	CT-scan measurement of axial callous volume of the dissected femora after day 14 of study Radiological evaluation of callous staging (based upon the grading system by Shuid et al. [19]) of the dissected femora after day 14 of study Radiological evaluation of fracture healing (based upon grading system by Warden et al. [20]) of the dissected femora after day 14 of study

Paskalev et al. [8]	Mixed-breed adult male dogs (control = 6; treatment = 6)	Diaphyses of right tibia and fibula (normal bone model)	Osteotomy	Open fracture	Internal fixation using plates	100 mg/day *α*-tocopherol (per oral)	30 days	Control group did not receive any treatment	Radiological evaluation of callous formation and bone remodeling staging of the operated limb on day 1, week 2, month 1, month 2, month 3, and month 4 after the surgery (the grading system was not stated)

Turk et al. [21]	Male Sprague-Dawley rats (control = 30; treatment = 30)	Right tibia (normal bone model)	Unspecified manual fracture procedure	Closed fracture	Internal fixation using Kirschner wires	20 mg/kg/day *α*-tocopherol (intraperitoneal)	60 days	Control groups received saline (intraperitoneal)	Radiological evaluation of the bone formation (based upon the grading system by Lane and Sandhu [16]) of the right tibia on day 60 of study Histopathological evaluation of the fracture healing (based upon the grading system by Allen et al. [22]) of the dissected right tibia after day 60 of study

Durak et al. [23]	New Zealand male white rabbits (Control = 10; Treatment = 10)	Right femur (Normal bone model)	Manual stress angulation	Closed fracture	External fixation using cast immobilization.	20 mg/kg *α*-tocopherol (intramuscular) 1 hour before and at the time of the procedure. 20 mg/kg/day *α*-tocopherol (intramuscular) after the procedure	5 days	Control group did not receive any treatment.	Histopathological evaluation of the fracture healing (based upon the grading system by Allen et al. [22]) of the dissected right tibia after day 21 of study.

Sarisözen et al. [24]	Adult Sprague-Dawley rats (control = 12; Vit. E only treatment = 12; Vit. C only treatment = 12; Vit. E and Vit. C combination treatment = 12)	Right femur (normal bone model)	Manual stress angulation	Closed fracture	Fracture left *in situ *	40 mg/kg *α*-tocopherol (intraperitoneal) daily from day 1 to day 3 and three times a week from day 4 onward	14 days 21 days	Control group did not receive any treatment Vit. C only group received 200 mg/kg vit. C daily (intraperitoneal) from day 1 to day 3 and three times a week from day 4 onward Vit. C and Vit. E combination group received 40 mg/kg vit. E and 200 mg/kg Vit. C daily (intraperitoneal) from day 1 to day 3 and three times a week from day 4 onward	Radiological evaluation of fracture healing (based upon the callous index calculation by Oni et al. [25]) of the right tibias from the rats sacrificed on day 14 and 21 Histopathological evaluation of fracture healing (based upon the grading system by Allen et al. [22]) of the dissected right tibias from the rats sacrificed on day 14 and 21

**Table 2:** Results of past animal studies on the effect of vitamin E on healing of bone fracture.

Study	Reported results			Model proposed for mechanism of action of vitamin E in study	Conclusion of effect of vitamin E based on the phase of fracture healing examined in study
	Reported descriptive values of main outcome measure(s)	Reported inferential statistical analysis	Reported conclusion
	(A)* Radiological-based grade of bone formation *[16]				
	(1) Day 20 Control group (*n* = 15): mean = 1.46, s.d. = 0.51 Treatment group (*n* = 15): mean = 1.73, s.d. = 0.45	Mann-Whitney *U* test: *P* > 0.05	The radiological-based bone formation grade of the treatment group was significantly higher than the control group on day 30 and day 40. There was no significant difference on day 20		
	(2) Day 30 Control group: mean (*n* = 15) = 3.46, s.d. = 0.83 Treatment group: mean (*n* = 15) = 4.60, s.d. = 0.91	Mann-Whitney *U* test: *P* < 0.01			
	(3) Day 40 Control group: mean (*n* = 15) = 5.46, s.d. = 0.83 Treatment group: mean (*n* = 15) = 7.73, s.d. = 1.09	Mann-Whitney *U* test: *P* < 0.01		The positive effect of antioxidant property of *α*-tocopherol (measured based on Total Antioxidant Capacity level) on secondary bone healing (intramembranous ossification) in normal bone	Favourable effect seen during ischemic phase (day 30), but not inflammatory phase of healing (day 5) in rabbits The mechanism model was reported to be supported by the results in the study
Kurklu et al. [15]	(B)* Ratio of osteoblastic activity (distracted/normal) *				
	(1) Day 5 Control group: mean (*n* = 15) = 0.97, s.d. = 0.18 Treatment group: mean (*n* = 15) = 1.06, s.d. = 0.20	Mann-Whitney *U* test: *P* > 0.05	The osteoblastic activity of the treatment group was significantly higher than the control group on day 20. There was no significant difference on day 5		
	(2) Day 20 Control group: mean (*n* = 15) = 1.65, s.d. = 0.54 Treatment group: mean (*n* = 15) = 3.63, s.d. = 1.06	Mann-Whitney *U* test: *P* < 0.01			
	(C)* Histological-based grade of bone formation* [17]		The histological-based bone formation grade of the treatment group was significantly higher than the control group and showed mature bone formation		

	(1) Day 40 Control group: mean (*n* = 15) = 8.00, s.d. = 0.92 Treatment group: mean (*n* = 15) = 9.86, s.d. = 0.35	Mann-Whitney *U* test: *P* < 0.01		
					
	(A)* Callous volume *				
	(1) Day 14 sham group (*n* = 8): mean = ±0.15, variability = ? (graph form only) Ovariectomized control group (*n* = 8): mean = ±0.17, variability = ? (graph form only) Ovariectomized treatment group (*n* = 8): mean = ±0.20, variability = ? (graph form only)	*P* > 0.05 (however, it is not known whether ANOVA or Kruskal-Wallis tests was used)	There was no significant difference in the callous volume between the three groups		
Shuid et al. (2011) [18]	(B)* Radiological-based * *score of callous staging * [19]			The boosting-up effect of *α*-tocopherol on antioxidant enzymes activities (measured based on Bone Superoxide Dismutase and Bone Glutathione Peroxidase levels) on secondary bone healing in osteoporotic bone	Favourable effect seen during early phase of healing (day 14) in rats The mechanism model was reported to be supported by the results in the study (only for bone superoxide dismutase)
	(1) Day 14 Sham group (*n* = 8): Score 1 (*n* = 1), Score 2 (*n* = 5), Score 3 (*n* = 2) Ovariectomized control group (*n* = 8): Score 2 (*n* = 2), Score 3 (*n* = 4), Score 4 (*n* = 2) Ovariectomized treatment group (*n* = 8): Score 2 (*n* = 2), Score 3 (*n* = 3), Score 4 (*n* = 3)	Pearson chi-square test: *P* > 0.05	There was no significant difference of callous staging grades between the three groups		
	(C)* Radiological-based score of fracture healing staging* [20]		Ovariectomized control group has significantly more Score 2 and less Score 3 of the fracture healing grade than both sham-operated and ovariectomized treatment group The fracture healing score of the ovariectomized treatment group was similar with the sham-operated group		
	(1) Day 14 Sham-operated group (*n* = 8): Score 2 (*n* = 1), Score 3 (*n* = 7) Ovariectomized control group (*n* = 8): Score 2 (*n* = 5), Score 3 (*n* = 3) Ovariectomized treatment group (*n* = 8): Score 2 (*n* = 1), Score 3 (*n* = 7)	Pearson chi-square test: *P* < 0.05 (difference between ovariectomized control group against sham-operated and ovariectomized treatment groups in terms of score)			

					
	(A) *Radiological-based * *evaluation of callous formation staging (unspecified) *		It was stated that callous formation stage is better in the treatment group compared to the control one		
Paskalev et al. [8]	(1) Day 1, week 2, month 1, month 2, month 3, and month 4 Control group (*n* = 6): no descriptive value reported Treatment group (*n* = 6): no descriptive value reported	No statistical analysis was reported		The boosting-up effect of *α*-tocopherol on antioxidant enzymes activities (measured based on Serum Catalase level) on primary bone healing in normal bone	Favourable effect of *α*-tocopherol given for 30 days postosteotomy seen from week 1 to the end of the third month of the study (i.e., from early to the late phase of healing) in dogs The mechanism model was reported to be supported by the results in the study
	(B) *Radiological-based * *evaluation of bone remodelling staging (unspecified) *		It was stated that bone remodeling stage is better in the treatment group compared to the control		
	(1) Day 1, week 2, month 1, month 2, month 3, and month 4 Control group (*n* = 6): no descriptive value reported Treatment group (*n* = 6): no descriptive value reported	No statistical analysis was reported			

	(A)* Radiological-based grade of bone formation* [16]		The radiological-based bone formation grade was significantly superior in the treatment group compared to the control group		
Turk et al. [21]	(1) Day 60: Control group (*n* = 10): Grade 3 (*n* = 9), Grade 4 (*n* = 1) Treatment group (*n* = 10) = Grade 3 (*n* = 1), Grade 4 (*n* = 9)	Pearson chi-square test: *P* < 0.05 (difference between control group and treatment group in terms of Grade 3 and Grade 4)		The positive effect of antioxidant property of *α*-tocopherol (measured based on Erythrocyte Malondialdehyde level) on secondary bone healing in normal bone	Favourable effect seen during early phase (day 15) up to late phase (day 60) of healing in rats The mechanism model was reported to be supported by the results in the study
	(B) *Histological-based grade of fracture healing* [22]		The histological-based bone formation grade was significantly superior in the treatment group compared to the control group		
	(1) Day 60: Control group (*n* = 10): Grade 2 (*n* = 1), Grade 3 (*n* = 7), Grade 4 (*n* = 2) Treatment group (*n* = 10) = Grade 3 (*n* = 1), Grade 4 (*n* = 9)	Pearson chi-square test: *P* < 0.05 (difference between control group and treatment group in terms of Grade 3 and Grade 4)			

Durak et al. [23]	(A) *Histological-based grade of fracture healing *[22]		The histological-based bone formation grade of the treatment group was significantly higher than the control group	No specific mechanism model was tested for the effect of *α*-tocopherol on secondary bone healing in normal bone	Favourable effect of *α*-tocopherol given for 5 days postfracture seen up to day 21 of study in rabbits (actual healing phase not stated)
	(1) Day 21 (16 days after the last treatment): Control group (*n* = 10): Grade 2 (*n* = 6), Grade 3 (*n* = 4) [mean = 2.4, s.d. = 0.5] Treatment group (*n* = 10) = Grade 2 (*n* = 1), Grade 3 (*n* = 5), Grade 4 (*n* = 4) [mean = 1.46, s.d. = 0.51]	Mann-Whitney *U* test: *P* < 0.01			
					
	(A)* Callous index *				
	(1) Day 14 Control group (*n* = 6): mean = 1.64, variability = not reported Vit. E only treatment group (*n* = 6): mean = 1.49, variability = not reported Vit. C only treatment group (*n* = 6): mean = 2.17, variability = not reported Vit. E and C combination treatment group (*n* = 6): mean = 1.88, variability = not reported	Kruskal-Wallis test: *P* < 0.05 Post-hoc test using Mann-Whitney *U* test: (a) *P* < 0.05 for comparison between Vit. C only group and combination group	On day 14, the mean callous index for the vitamin C only group was significantly higher than the combination group, whereas the vitamin E only group was the lowest and was similar to the control group On day 21, the mean callous index for the vitamin C only group was significantly higher than all groups, whereas the vitamin E only group was higher (although not significant) than the combination and control groups		
	(2) Day 21 Control group (*n* = 6): mean = 1.90, variability = not reported Vit. E only treatment group (*n* = 6): mean = 2.37, variability = not reported Vit. C only treatment group (*n* = 6): mean = 2.54, variability = not reported Vit. E and C combination treatment group (*n* = 6): mean = 1.93, variability = not reported	Kruskal-Wallis test: *P* < 0.05 Posthoc test using Mann-Whitney *U* test: (a) *P* < 0.05—Vit. C only group versus control group (b) *P* < 0.05—Vit. C only group vs. Vit. E only group (c) *P* < 0.05—Vit. C only group versus combination group			
Sarisözen et al. [24]	(B)* Histological-based grade of fracture healing *[22]			No specific mechanism model was tested for the effect of *α*-tocopherol on secondary bone healing in normal bone	No favourable effect seen during the early phase (day 14) of healing in rats
	(1) Day 14 Control group (*n* = 6): mean = 1.67, variability = not reported Vit. E only treatment group (*n* = 6): mean = 1.50, variability = not reported Vit. C only treatment group (*n* = 6): mean = 2.67, variability = not reported Vit. E and C combination treatment group (*n* = 6): mean = 2.16, variability = not reported	Kruskal-Wallis test: *P* < 0.05 Posthoc test using Mann-Whitney *U* test: (a) *P* < 0.05—Vit. C only group versus control group. (b) *P* < 0.05—Vit. C only group versus Vit. E only group. (c) *P* < 0.05—Combination group versus control group. (d) *P* < 0.05—Combination group versus Vit. E only group	The mean grade for the vitamin C only group and combination group was significantly higher than the vitamin E only group and control group on day 14 and day 21. On day 14, the mean grade for the vitamin E only group was lower (although not significant) than the control group, whereas, on day 21, the mean grade for the vitamin E only group was higher (although not significant) than the control group		
					
	(2) Day 21 Control group (*n* = 6): mean = 2.00, variability = not reported Vit. E only treatment group (*n* = 6): mean = 2.33, variability = not reported Vit. C only treatment group (*n* = 6): mean = 3.17, variability = not reported Vit. E and C combination treatment group (*n* = 6): mean = 3.00, variability = not reported	Kruskal-Wallis test: *P* < 0.05 Posthoc test using Mann-Whitney *U* test: (a) *P* < 0.05—Vit. C only group versus control group. (b) *P* < 0.05—Vit. C only group versus Vit. E only group (c) *P* < 0.05—Combination group versus control group (d) *P* < 0.05—Combination group versus Vit. E only group			
					

**Table 3:** Quality of methods and results reported in animal studies included in the review (based upon ARRIVE Guidelines).

Section	Item in ARRIVE	ARRIVE Guidelines recommendation	Status in article (if described: +; if not described satisfactorily: commentary was given)					
			Kurklu et al. [15]	Shuid et al. [18]	Paskalev et al. [8]	Turk et al. [21]	Durak et al. [23]	Sarisözen et al. [24]
Methods								

Ethical statement	5	Indicate the nature of the ethical review permissions, relevant licences (e.g., Animal (Scientific Procedures) Act 1986), and national or institutional guidelines for the care and use of animals, that cover the research	+	+	+	+	+	+

Study design	6	For each experiment, give brief details of the study design including the following						
	6a	The number of experimental and control groups	+	+	+	+	+	+
	6b	Any steps taken to minimise the effects of subjective bias when allocating animals to treatment (e.g., randomisation procedure) and when assessing results (e.g., if performed, describe who was blinded and when)	+	Randomization was not described for the allocation of sham group	Not described	No blinding procedure was described	Not described	Not described
	6c	The experimental unit (e.g., a single animal, group, or cage of animals)	+	+	+	+	+	+

Experimental procedures	7	For each experiment and each experimental group, including controls, provide precise details of all procedures carried out. For example:						
	7a	How (e.g., drug formulation and dose, site and route of administration, anaesthesia and analgesia used (including monitoring), surgical procedure, method of euthanasia). Provide details of any specialist equipment used, including supplier(s)	The full formulation of *α*-tocopherol was not described	Method of euthanasia was not described The supplier of the fracture device was not reported	+	Method of euthanasia was not described	+	Methods of analgesia and euthanasia were not described
	7b	When (e.g., time of day)	Not described	Not described	Not described	Not described	Not described	Not described
	7c	Where (e.g., home cage, laboratory, water maze)	+	+	Not described	+	+	Not described
	7d	Why (e.g., rationale for choice of specific anaesthetic, route of administration, drug dose used)	Not described	Not described	+	Not described	Not described	Not described

Experimental animals	8							
	8a	Provide details of the animals used, including species, strain, sex, developmental stage (e.g., mean or median age plus age range), and weight (e.g. mean or median weight plus weight range)	Sex and age were not described	Age was not described	+	Age was not described	Age was not described	Sex was not described
	8b	Provide further relevant information such as the source of animals, international strain nomenclature, genetic modification status (e.g., knock-out or transgenic), genotype, health/immune status, drug or test naïve, previous procedures, and so forth	Not described	Not described	+	Not described	Not described	Not described

Housing and husbandry	9	Provide details of						
	9a	Housing (type of facility e.g., specific pathogen free (SPF); type of cage or housing; bedding material; number of cage companions; tank shape and material, etc., for fish).	Cage type was not fully described	Cage type was not fully described	Cage type was not fully described	Cage type was not fully described	Cage type was not fully described	Cage type was not fully described
	9b	Husbandry conditions (e.g., breeding programme, light/dark cycle, temperature, quality of water, etc., for fish, type of food, access to food, and water, environmental enrichment)	Specific type of food given was not described	+	Light/dark cycle and temperature were not described	Specific type of food given was not described	Not described (only access to food was described)	Not described (only access to food and water was described)
	9c	Welfare-related assessments and interventions that were carried out prior to, during, or after the experiment	Not described	+	+	Not described	Analgesia was not given after the fracture procedure (just depended on cast immobilization)	Not described

Sample size	10							
	10a	Specify the total number of animals used in each experiment, and the number of animals in each experimental group	+	+	+	+	+	+
	10b	Explain how the number of animals was arrived at. Provide details of any sample size calculation used	Not described	Not described	Not described	Not described	Not described	Not described
	10c	Indicate the number of independent replications of each experiment, if relevant	Not described	Not described	Not described	Not described	Not described	Not described
	11							
Allocating animals to experimental groups	11a	Give full details of how animals were allocated to experimental groups, including randomisation or matching if performed	Randomization procedure was not fully explained	Randomization procedure was not fully explained for the allocation of treatment groups	Not explained	Randomization procedure was not fully explained	Not explained	Not explained
	11b	Describe the order in which the animals in the different experimental groups were treated and assessed	Not described	Not described	Not described	Not described	Not described	Not described

Experimental outcomes	12	Clearly define the primary and secondary experimental outcomes assessed (e.g., cell death, molecular markers, behavioural changes)	+	+	+	+	+	+

Statistical methods	13							
	13a	Provide details of the statistical methods used for each analysis	+	+	+	+	+	+
	13b	Specify the unit of analysis for each dataset (e.g., single animal, group of animals, single neuron)	+	+	+	+	+	+
	13c	Describe any methods used to assess whether the data met the assumptions of the statistical approach	+ Not relevant as nonparametric test was used	+ Not relevant as nonparametric test was used	+ Not relevant as nonparametric test was used	+ Not relevant as nonparametric test was used	+ Not relevant as nonparametric test was used	+ Not relevant as nonparametric test was used
Results								

Baseline data	14	For each experimental group, report relevant characteristics and health status of animals (e.g., weight, microbiological status and drug or test naive) prior to treatment or testing (this information may often be tabulated)	Not fully described (i.e., only baseline weight was reported)	Not fully described (i.e., only baseline weight was reported)	Not fully described (i.e., only baseline weight was reported)	Not fully described (i.e., only baseline weight was reported)	Not fully described (i.e., only baseline weight was reported)	Not fully described (i.e., only baseline weight was reported)

Numbers analysed	15							
	15a	Report the number of animals in each group included in each analysis. Report absolute numbers (e.g., 10/20, not 50%)	+	+	Not described	+	+	Not described
	15b	If any animals or data were not included in the analysis, explain why	? (no exclusion was reported)	? (no exclusion was reported)	? (no exclusion was reported)	? (no exclusion was reported)	? (no exclusion was reported)	? (no exclusion was reported)

Outcomes and estimation	16	Report the results for each analysis carried out, with a measure of precision (e.g., standard error or confidence interval)	+ (see full commentary in reanalysis table)	Some statistical analyses were not reported fully (see full commentary in reanalysis table)	Not described. (see full commentary in reanalysis table)	+ (see full commentary in reanalysis table)	+ (see full commentary in reanalysis table)	The measure of precision was not reported in the result (see full commentary in reanalysis table)

	17							
Adverse events	17a	Give details of all important adverse events in each experimental group	+ (no complication reported)	Not described	Not described	Not described	Not described	Not described
	17b	Describe any modifications to the experimental protocols made to reduce adverse events	Not described	Not described	Not described	Not described	Not described	Not described

Total items described satisfactorily		Methods section (per 22 items) Results section (per 6 items)	10 3	11 1	13 0	9 2	10 2	8 0

**Table 4:** Commentary of statistical analysis and reanalysis of results from past animal studies of the effect of vitamin E on bone fracture healing.

Study	Result	Reanalysed result (where necessary or possible) and final conclusion (focusing on the vitamin E group versus control group)	Effect size and power of the result via G*Power (vitamin E group versus control group)	Sample size for future replication of results via G*Power
	(A) *Radiological-based grade of bone formation *			
		*Reanalysis was unnecessary for conclusion *	If *α* = 0.05, 2-tailed; distribution: minimum ARE Effect size, *d* = 0.56 Power = 0.28	If *α* = 0.05, 2-tailed; power = 0.80; distribution: minimum ARE Sample size for each group (equal number) = 59
	(1) Day 20 analysis	Mann-Whitney *U* test: *P* > 0.05		
		Final conclusion: there was no significant difference between the treatment group and control group in terms of radiological-based grade of bone formation		
		*Reanalysis was unnecessary for conclusion *	If *α* = 0.05, 2-tailed; distribution: minimum ARE Effect size, *d* = 1.31 Power = 0.89	If *α* = 0.05, 2-tailed; power = 0.80; distribution: minimum ARE Sample size for each group (equal number) = 12
	(2) Day 30 analysis	Mann-Whitney *U* test: *P* < 0.01		
		Final conclusion: the treatment group has significantly higher radiological-based grade of bone formation compared to control group		
		*Reanalysis was unnecessary for conclusion *	If *α* = 0.05, 2-tailed; distribution: minimum ARE Effect size, *d* = 2.34 Power ≈ 1.00	If *α* = 0.05, 2-tailed; power = 0.80; distribution: minimum ARE Sample size for each group (equal number) = 5
	(3) Day 40 analysis	Mann-Whitney *U* test: *P* < 0.01		
Kurklu et al. [15]		Final conclusion: the treatment group has significantly higher radiological-based grade of bone formation compared to control group		
	(B) *Ratio of osteoblastic activity *			
	(1) Day 5 analysis	*Reanalysis was unnecessary for conclusion *	If *α* = 0.05, 2-tailed; distribution: minimum ARE Effect size, *d* = 0.47 Power = 0.21	If *α* = 0.05, 2-tailed; power = 0.80; distribution: minimum ARE Sample size for each group (equal number) = 83
		Mann-Whitney *U* test: *P* > 0.05		
		Final conclusion: there was no significant difference between the treatment group and control group in terms of osteoblastic activity		
	(2) Day 20 analysis	*Reanalysis was unnecessary for conclusion *	If *α* = 0.05, 2-tailed; distribution: minimum ARE Effect size, *d* = 2.35 Power ≈ 1	If *α* = 0.05, 2-tailed; power = 0.80; distribution: minimum ARE Sample size for each group (equal number) = 5
		Mann-Whitney *U* test: *P* < 0.01		
		Final conclusion: the treatment group has significantly higher osteoblastic activity compared to control group		
	(C) *Histological-based grade of bone formation *			
	(1) Day 40 analysis	*Reanalysis was unnecessary for conclusion *	If *α* = 0.05, 2-tailed; distribution: minimum ARE Effect size, *d* = 2.67 Power ≈ 1	If *α* = 0.05, 2-tailed; power = 0.80; distribution: minimum ARE Sample size for each group (equal number) = 5
		Mann-Whitney *U* test: *P* < 0.01		
		Final conclusion: the treatment group has significantly higher histological-based grade of bone formation compared to control group		
				

	(A) *Callous volume *			
		*Not enough information from the article for reanalysis *	Not enough information from the article for calculation	Not enough information from the article for calculation
	(1) Day 14 analysis	Unknown statistical test: *P* > 0.05		
		Final conclusion: There was no significant difference of callous volume between the ovariectomized treatment group and ovariectomized control group		
	(B)* Radiological-basedscore of callous staging *			
		*Reanalysis was performed using the tabulated data reported for the chi-square test in the article *		
		Descriptive results:		
		(1) Sham group (*n* = 8): median = 2 (mean = 2.12, s.d. = 0.64)		
		(2) Ovariectomized control group (*n* = 8): median = 3 (mean = 3.00, s.d. = 0.76)		
	(1) Day 14 analysis	(3) Ovariectomized treatment group (*n* = 8): median = 3 (mean = 3.12, s.d. = 0.84)	If *α* = 0.05, 2-tailed; distribution: minimum ARE Effect size, *d* = 0.15 Power = 0.06	If *α* = 0.05, 2-tailed; power = 0.80; distribution: minimum ARE Sample size for each group (equal number) = 811
		Kruskal-Wallis test: *H*(df = 2) = 6.5, *P* < 0.05		
		Posthoc Mann-Whitney *U* test focused on the comparison between ovariectomized treatment group and ovariectomized control group (Bonferroni's correction was not necessary in this case): *U* = 29, *P* > 0.05		
Shuid et al. [18]		Final conclusion: there was no significant difference between the treatment group and control group in terms of radiological-based callous staging		
	(C)* Radiological-based score of fracture healing staging *			
	(1) Day 14 analysis	*Reanalysis was performed using the tabulated data reported for the chi-square test in the article *		
		Descriptive results		
		(1) Sham group (*n* = 8): median = 3 (mean = 2.88, s.d. = 0.35)		
		(2) Ovariectomized control group (*n* = 8): median = 2 (mean = 2.38, s.d. = 0.52)		
		(3) Overaiectomized treatment group (*n* = 8): median = 3 (mean = 2.88, s.d. = 0.35)	If *α* = 0.05, 2-tailed; distribution: minimum ARE Effect size, *d* = 1.13 Power = 0.49	If *α* = 0.05, 2-tailed; power = 0.80; distribution: minimum ARE Sample size for each group (equal number) = 16
		Kruskal-Wallis test: *H*(df = 2) = 6.2, *P* < 0.05		
		Post-hoc Mann-Whitney *U* test focused on the comparison between ovariectomized treatment group and ovariectomized control group (Bonferroni's correction was not necessary in this case): *U* = 16, *P* < 0.05		
		Final conclusion: the treatment group had significantly higher radiological-based fracture healing staging than control group		
				

	(A)* Radiological-based * *evaluation of callous formation staging (unspecified) *			
Paskalev et al. [8]	(1) Day 1, week 2, month 1, month 2, month 3, and month 4 analysis	*Not enough information from the article for reanalysis* No statistical test result reported Final conclusion: it was stated that callous formation stage was better in the treatment group compared to the control	Not enough information from the article for calculation	Not enough information from the article for calculation
	(B)* Radiological-based * *evaluation of bone remodeling staging (unspecified) *			
	(1) Day 1, week 2, month 1, month 2, month 3, and month 4 analysis	*Not enough information from the article for reanalysis* No statistical test result reported Final conclusion: it was stated that bone remodeling stage was better in the treatment group compared to the control	Not enough information from the article for calculation	Not enough information from the article for calculation

	(A)* Radiological-based grade of bone formation *			
	(1) Day 60 analysis	*Reanalysis was performed using the tabulated data reported for the chi-square test in the article *	If *α* = 0.05, 2-tailed; distribution: minimum ARE Effect size, *d* = 2.5 Power ≈ 1	If *α* = 0.05, 2-tailed; power = 0.80; distribution: minimum ARE Sample size for each group (equal number) = 5
		Descriptive results		
		Control group (*n* = 10): median = 3 (mean = 3.10, s.d. = 0.32)		
		Treatment group (*n* = 10): median = 4 (mean = 3.90, s.d. = 0.32)		
		Mann-Whitney *U* test: *U* = 10.0, *P* < 0.01		
		Final conclusion: the treatment group had significantly higher radiological-based grade of bone formation than control group		
Turk et al. [21]	(B)* Histological-based grade of fracture healing *			
	(1) Day 60 analysis	*Reanalysis was performed using the tabulated data reported for the chi-square test in the article *	If *α* = 0.05, 2-tailed; distribution: minimum ARE Effect size, *d* = 2.5 Power ≈ 1	If *α* = 0.05, 2-tailed; power = 0.80; distribution: minimum ARE Sample size for each group (equal number) = 5
		Descriptive results		
		Control group (*n* = 10): median = 3 (mean = 3.10, s.d. = 0.57)		
		Treatment group (*n* = 10): median = 4 (mean = 3.90, s.d. = 0.32)		
		Mann-Whitney *U* test: *U* = 14.5, *P* < 0.01		
		Final conclusion: the treatment group had significantly higher histological-based grade of bone formation than control group		
				

	(A)* Histological-based grade of fracture healing *			
	(1) Day 21 (16 days after the last treatment) analysis	*Reanalysis was performed using the tabulated data reported for the chi-square test in the article *	If *α* = 0.05, 2-tailed; distribution: minimum ARE Effect size, *d* = 1.49 Power = 0.82	If *α* = 0.05, 2-tailed; power = 0.80; distribution: minimum ARE Sample size for each group (equal number) = 10
		Descriptive results:		
Durak et al. [23]		Control group (*n* = 10): median = 2 (mean = 2.40, s.d. = 0.52)		
		Treatment group (*n* = 10): median = 3 (mean = 3.30, s.d. = 0.68)		
		Mann-Whitney *U* test: *U* = 17, *P* < 0.01		
		Final conclusion: the treatment group had significantly higher histological-based grade of fracture healing than control group		

	(A)* Callous index *			
		*Reanalysis was unnecessary for conclusion *	Not enough information from the article for calculation (absence of standard deviation value)	Not enough information from the article for calculation (absence of standard deviation value)
	(1) Day 14 analysis	Posthoc Mann-Whitney *U* test: *P* > 0.05		
		Final conclusion: there was no significant difference between the Vit. E only treatment group and control group in terms of callous index		
		*Reanalysis was unnecessary for conclusion *	Not enough information from the article for calculation (absence of standard deviation value)	Not enough information from the article for calculation (absence of standard deviation value)
	(2) Day 21 analysis	Post-hoc Mann-Whitney *U* test: *P* > 0.05		
		Final conclusion: there was no significant difference between the Vit. E only treatment group and control group in terms of callous index		
Sarisözen et al. [24]	(B)* Histological-based grade of fracture healing *			
		*Reanalysis was unnecessary for conclusion *	Not enough information from the article for calculation (absence of standard deviation value)	Not enough information from the article for calculation (absence of standard deviation value)
	(1) Day 14 analysis	Post-hoc Mann-Whitney *U* test: *P* > 0.05		
		Final conclusion: there was no significant difference between the Vit. E only treatment group and control group in terms of histological-based grade of fracture healing		
		*Reanalysis was unnecessary for conclusion *	Not enough information from the article for calculation (absence of standard deviation value)	Not enough information from the article for calculation (absence of standard deviation value)
	(2) Day 21 analysis	Post-hoc Mann-Whitney *U* test: *P* > 0.05		
		Final conclusion: there was no significant difference between the Vit. E only treatment group and control group in terms of histological-based grade of fracture healing		

**Table 5:** Meta-synthesis based upon the cross-tabulation of individual parameter results from the included studies in the review according to type of procedure, type of parameter observed and day of post-fracture.

Type of parameter observed	Type of procedure	The result showed that the vitamin E treatment group was significantly superior to the control group	Postfracture day					
			Below day 10	Day 10–19	Day 20–29	Day 30–39	Day 40–49	Day 50 and above
Radiological-based assessment of bone formation	Internal fixation with Kirschner wire	Yes		1^B^ *d* = 1.13*				1^C^ *d* = 2.50
		No						
	Distraction using external fixator	Yes				1^A^ *d* = 1.31	1^A^ *d* = 2.34	
		No			1^A^ *d* = 0.56			

Histological-based assessment of bone formation	Internal fixation with Kirschner wire	Yes						1^C^ *d* = 2.5
		No						
	Distraction using external fixator	Yes					1^A^ *d* = 2.67	
		No						
	External fixation using cast immobilization	Yes			1^D^ *d* = 1.49			
		No						
	Fracture left *in situ *	Yes						
		No		1^E^ *d* = ?	1^E^ *d* = ?			

Radiological-based assessment of callous volume/callous index	Internal fixation with Kirschner wire	Yes						
		No		1^B^ *d* = ?				
	Fracture left *in situ *	Yes						
		No		1^E^ *d* = ?	1^E^ *d* = ?			

Radiological-based assessment of callous formation	Internal fixation with Kirschner wire	Yes						
		No		1^B^ *d* = 0.15				
							

Osteoblastic activity	Distraction using external fixator	Yes			1^A^ *d* = 2.35			
		No	1^A^ *d* = 0.47					

^A^Result from Kurklu et al. [15]: normal bone, rabbit model, 20 mg/kg/day (intramuscular).

^B^Result from Shuid et al. [18]: osteoprotic bone, rat model, 60 mg/kg/day (per oral).

^C^Result from Turk et al. [21]: normal bone, rat model, 20 mg/kg/day (intraperitoneal).

^D^Result from Durak et al. [23]: normal bone, rabbit model, 20 mg/kg/day (intramuscular) for the initial 5 days only.

^E^Result from Sarisözen et al. [24]: normal bone, rat model, 40 mg/kg (intraperitoneal) (daily: day 1 to 3; 3x per week: day 4 onward).

*Significant result which has power less than 0.8.

## List of Abbreviations

MEDLINE: Medical Literature Analysis and Retrieval System Online
CINAHL: Cumulative Index to Nursing and Allied Health Literature
ARRIVE: Animal in Research: Reporting In Vivo Experiments
SPSS: Statistical Package for the Social Sciences
ARE: Minimum Asymptomatic Relative Efficiency
*P*: The calculated probability value of type 1 error
*α*: The probability value of type 1 error where the significance level is set
*d*: Cohen's standardized effect size, *d*
Kg: kilogram
mg: Milligram
*n*: Sample size
vit.: Vitamin.
